# Effects of the COVID-19 Pandemic on Brief Resolved Unexplained Events (BRUEs) in Children: A Comparative Analysis of Pre-Pandemic and Pandemic Periods

**DOI:** 10.3390/life14030392

**Published:** 2024-03-15

**Authors:** Luana Nosetti, Marco Zaffanello, Giorgio Piacentini, Francesca De Bernardi, Cristina Cappelluti, Camilla Sangiorgio, Massimo Agosti

**Affiliations:** 1 Pediatric Sleep Disorders Center, Division of Pediatrics, F. Del Ponte Hospital, Insubria University, 21100 Varese, Italy; luana.nosetti@uninsubria.it (L.N.);; 2 Department of Surgery, Dentistry, Pediatrics and Gynecology, University of Verona, 37100 Verona, Italy; 3 Division of Otorhinolaryngology, ASST Settelaghi, Department of Biotechnology and Life Sciences, University of Insubria, 21100 Varese, Italy; francesca.debernardi@asst-settelaghi.it; 4 Department of Medicine and Surgery, University of Insubria, 21100 Varese, Italy; massimo.agosti@uninsubria.it

**Keywords:** apparent life-threatening events, brief resolved unexplained events, COVID-19, infant, newborn, polysomnography, pregnancy, SARS-CoV-2

## Abstract

Background: Brief Resolved Unexplained Events (BRUEs), formerly known as Apparent Life-Threatening Events (ALTEs), are concerning episodes of short duration (typically <1 min) characterized by a change in breathing, consciousness, muscle tone, and/or skin color. In some cases, SARS-CoV-2 infection has been associated with episodes of BRUEs in previously healthy children. This study aimed to compare the demographic, respiratory, perinatal, and infectious characteristics in children affected by BRUEs before the COVID-19 pandemic and after the spread of SARS-CoV-2. Methods: We conducted a retrospective observational study covering January 2018 to March 2020 (pre-COVID-19) and April 2023 (during the ongoing COVID-19 pandemic). Collected variables included clinical information during pregnancy and neonatal details of children with BRUEs. Results: The number of children in the pre-COVID-19 period was 186 (41%); after the emergence and spread of SARS-CoV-2 this number was 268 (59%). The risk of infection at birth for children developing BRUEs was higher during the pandemic. Children were less likely to have ongoing symptomatic infection during BRUEs during the pandemic (coefficient B = 0.783; *p* = 0.009). Respiratory symptoms during BRUEs were more frequent during the pandemic (coefficient B = 0.654; *p* = 0.052). Fever during BRUEs was less likely during the pandemic (coefficient B = −0.465, *p* = 0.046). Conclusions: These findings could have significant clinical implications for managing children with BRUEs during the COVID-19 pandemic.

## 1. Introduction

Brief Resolved Unexplained Events (BRUEs), formerly known as Apparent Life-Threatening Events (ALTEs), are concerning episodes of short duration (typically < 1 min) characterized by a change in breathing, consciousness, muscle tone (hyper- or hypo-tonia), and/or skin color (cyanosis or pallor) [1,2]. The American Academy of Pediatrics (AAP) introduced the term BRUE to describe episodes in which a child exhibits one or more of the following symptoms: apnea (breathlessness), cyanosis (change in skin color to blue or purple), alteration of muscle tone, or alteration in response to the environment [3]. Italian guidelines distinguish between BRUEs and ALTEs, reserving the latter for severe cases [4].

The incidence of BRUEs varies in the literature. Prospective studies indicate an incidence ranging from 0.58 to 2.46 per 1000 live births [4]. The most common diagnosis (63%) was no diagnosis. Of the 37% who were discharged with a diagnosis, the most frequent diagnoses were gastroesophageal reflux and choking/gagging. Only 4.1% of the diagnoses were classified as severe [2,5].

Identifying the causes of BRUEs is a challenge. About 39.1% of BRUEs have been attributed to lower respiratory tract infections [6]. Other associated causes include respiratory tract anomalies [5]. For infants who have experienced a BRUE, respiratory infection may be considered if there is a fever or persistent respiratory symptoms [1,4].

Viruses most frequently implicated in BRUEs are respiratory syncytial virus (RSV), influenza viruses, and parainfluenza viruses [4,7]. In some BRUE cases, an infection with the HCoV-229E coronavirus may be an underlying cause [8]. Clinical cases have been reported, including a previously healthy infant infected with the SARS-CoV-2 virus at 8 months of age developing recurrent episodes of BRUE [9], and another previously healthy infant experiencing a severe episode of a BRUE [10]. A case has been described of a healthy 2-week-old newborn with apnea and coinfection with SARS-CoV-2 and influenza B [11].

This study aims to compare the demographic, respiratory, perinatal, and infectious characteristics of children affected by BRUEs before the SARS-CoV-2 pandemic and after the onset and spread of the virus. The primary objective is to contribute to identifying any impacts that occurred after the emergence of SARS-CoV-2 and assess whether there have been variations in the manifestation of such events with the spread of the virus.

## 2. Materials and Methods

The study was conducted at the sudden infant death syndrome (SIDS) Center of the Lombardy Region and the Center for Sleep Respiratory Disorders and Pediatric Pulmonology at ‘F. Del Ponte’ Hospital, University of Insubria, located in Varese, Italy (ZIP code: 21100). The SIDS Center specializes in the study of (SIDS), known as “crib death”. It is one of the few centers in Italy dedicated to SIDS research.

### 2.1. Study Population

We reviewed the medical records of newborns admitted to our hospital for BRUE. The period under consideration ranges from January 2018 to March 2020 (pre-COVID-19) and extends from April 2020 to April 2023, considering the onset and spread of SARS-CoV-2. Inclusion criteria were children aged between 1 month and 2 years. We applied the Italian guidelines for diagnosing BRUE [4].

### 2.2. Data Extraction from Medical Records

Data extraction from medical records included information related to the newborn [birth weight, age, gender, date of BRUE], variables related to pregnancy [infection during pregnancy, SARS-CoV-2 infections during pregnancy, other infections during pregnancy, infectious risk at birth], variables associated with symptoms and contagious agents [respiratory symptoms, fever, identified infectious agent: SARS-CoV-2, respiratory syncytial virus (RSV), rhinovirus, group B streptococcus (GBS)], variables related to monitoring parameters (AHI, minimum SpO_2_, average SpO_2_), and variables related to clinical signs [abnormal breathing, desaturations, short apneas, prolonged apneas (>15 s), and periodic breathing].

### 2.3. Nap Polysomnography

Upon arrival, newborns underwent a polysomnography (PSG) during their naps. PSG was conducted using a Compumedics P/L E-series instrument based in Melbourne, Australia. The device recorded nasal flow pressure (measured via nasal cannulas), nasal flow (via a thermistor), thoracic and abdominal movement (using Compumedics P/L inductive bands), oxygen saturation (SpO_2_) at a sampling rate of 1 sample per second, and electrocardiogram (ECG) with a sampling rate set at 500 Hz. Sleep staging was based on electroencephalogram (EEG) data. The sleep device included video and audio recordings and a position sensor. Nap PSG was conducted between two feeding sessions, starting no sooner than 30 min after the last meal [12]. The infant lay on their back in their bed. If the recording was less than 2 h, the study continued until the next feeding session. Upon discharge, newborns were equipped with home monitoring of apnea events using a portable recording device (VitaGuard^®^ V 3100, Getemed Medizin, Teltow, Germany).

### 2.4. Statistical Analysis

We performed statistical analysis using SPSS Statistics 22.0^®^ software (SPSS Inc., Chicago, IL, USA) for Windows (Microsoft Corp., Redmond, WA, USA). Measures included the Total Number of Cases, Mann–Whitney U, Wilcoxon W, Test Statistic, Standard Error, Standardized Test Statistic, and Asymptotic Significance of the two-way test. Compared categorical variables are expressed in terms of percentage frequencies. The Chi-square test (Pearson) assesses the association between variables, with corresponding asymptotic significance (two-sided). Binary logistic regression was used to examine the contribution of independent variables in predicting the presence or absence of a specific event or outcome. The statistical significance level was set at *p* < 0.05.

### 2.5. Ethical Aspects

The study was conducted according to the guidelines of the Declaration of Helsinki, and was approved by the Ethics Committee of ASST Sette Laghi, Ospedale di Circolo Fondazione Macchi, Varese, Italy (2016-187, 2017-110: 5 September 2017). The anonymized data (L.N., C.C., C.S.) were collected and stored in an Excel database on a password-protected PC. The anonymized data were subsequently analyzed by a single operator (M.Z.). In a retrospective study like this, informed consent does not apply.

## 3. Results

In total, the amount of enrolled children was 454 (53.7% males). The number of children in the pre-SARS-CoV-2 period was 186 (41%), after the emergence and spread of SARS-CoV-2, this number was 268 (59%).

The population treated did not change before and after COVID spread (Supplementary Table S1). No differences in the measured variables were observed between the children affected by BRUEs during the pre-SARS-CoV-2 period and the group after the onset and spread of SARS-CoV-2. Birth weight showed an average of 3094 ± 609 g. In the group of children who developed BRUEs after the beginning of SARS-CoV-2, the mean was 3,072 ± 643 g. The two-way test did not reveal significant differences between the groups (*p* = 0.588). In the pre-SARS-CoV-2 group, the mean age was 101 ± 101 days. In the group of children who developed BRUEs after the onset of SARS-CoV-2, the mean age decreased to 98.5 ± 84.3 days. The two-way test did not detect significant differences between the groups (*p* = 0.764). In the pre-SARS-CoV-2 group, the average monitoring duration was 124 ± 148 h. In the group of children who developed BRUEs after the onset of SARS-CoV-2, the average time was 106 ± 100 h. The two-way test did not find significant differences between the groups (*p* = 0.128).

Table 1 provides an overview of the dichotomous variables and their respective percentage values in a sample of 454 cases, which includes both pre-COVID-19 cases (*n* = 186) and post-SARS-CoV-2 cases (*n* = 268). The highest percentages were for ongoing symptomatic infection in BRUEs: 75.8%, short apnea: 76.4%, and pathologic breathing during BRUEs: 74%. The lowest percentages were for COVID-19 detected in BRUEs: 0.4%, GBS detected in BRUEs: 0.4%, and long apnea (yes): 0.4%. No CMV infection was found in pregnancy: 0%.

Table 2 presents the results of the binary logistic regression, where the dependent categorical variable corresponds to the two periods: the pre-COVID-19 period and the period of emergence and spread of SARS-CoV-2. Mothers of children with BRUEs have shown a lower risk of infection during pregnancy in the period of SARS-CoV-2 spread (coefficient B = −1.839; *p* = 0.001). The infectious risk at birth of children who will develop BRUE is higher in the period of SARS-CoV-2 spread (coefficient B = 0.783; *p* = 0.009). Asymptomatic infection during BRUEs was less likely during the SARS-CoV-2 spread (coefficient B = −0.413; *p* = 0.092). Respiratory symptoms during BRUEs were more frequent during the SARS-CoV-2 spread (coefficient B = 0.654; *p* = 0.052). Fever during BRUEs was less likely during the SARS-CoV-2 spread (coefficient B = −0.465, *p* = 0.046). There is a 37.2% decrease in fever during the SARS-CoV-2 spread, keeping all other variables constant. Finally, the average minimum SpO_2_ was less severe or higher during the SARS-CoV-2 spread (coefficient B = 0.055, *p* < 0.001). In addition, Table 2 presents the binary logistic regression results, where the dependent categorical variable corresponds to symptomatic infection, respiratory symptoms, or fever in the child with BRUEs. In particular, the probability of symptomatic disease during the spread of SARS-CoV-2 is about 37.1% less compared to the pre-COVID-19 period (*p* = 0.048), the possibilities of respiratory symptoms during the spread of SARS-CoV-2 are about 82.4% more compared to the pre-COVID-19 period (*p* = 0.070), and the probabilities of fever during the spread of SARS-CoV-2 are about 39.6% less compared to the pre-COVID-19 period (*p* = 0.034).

## 4. Discussion

This study conducted an in-depth examination of cases of children hospitalized following an episode of BRUE, comparing the period before the arrival of COVID-19 with that following the spread of SARS-CoV-2. These results could have important implications for understanding the impact of the COVID-19 pandemic on maternal and child health and the clinical management of BRUE. The fact that mothers of children with BRUEs have shown a lower risk of infection during pregnancy during the period of SARS-CoV-2 spread could suggest that the protective measures adopted during the pandemic (such as social distancing, the use of masks and hand hygiene) may have reduced the risk of exposure to pathogens.

Before exploring the implications of SARS-CoV-2 infection during pregnancy, it is essential to consider the overall picture. In most cases, women who received a diagnosis of SARS-CoV-2 during pregnancy had favorable pregnancy outcomes [13]. This could be partly due to the protective measures adopted during the pandemic, which reduced exposure to pathogens and the risk of infection during pregnancy.

Even though most pregnancies in women diagnosed with SARS-CoV-2 have favorable outcomes, exposure to SARS-CoV-2 during pregnancy could pose a higher risk of mortality, spontaneous abortion, preterm birth and low birth weight [14], pneumonia and thromboembolic disease [15], and admission to intensive care [16,17]. These factors could affect the health of newborns and potentially increase the risk of BRUEs.

Maternal infection with SARS-CoV-2 induces a fetal immune response even without placental infection or symptoms in the newborn [18,19]. The SARS-CoV-2 virus seems to primarily infect syncytiotrophoblast cells and other cells of the maternal–fetal interface. Fetuses or newborns with SARS-CoV-2 infection may present involvement of various organs 2021 [19]. It is unknown whether SARS-CoV-2 infection during pregnancy is directly related to an increased risk of BRUE. Therefore, it is crucial to consider that conditions associated with the infection could theoretically contribute to respiratory problems or other disorders in neonatal health, potentially raising the risk of BRUEs.

In this context, it is essential to underline that viral infections and pertussis are among the possible etiological causes of BRUEs [20]. In parallel, univariate BRUE predictors include prematurity, resuscitation attempt, apnea, cyanosis, and upper respiratory tract infection [21]. This intricate network of factors requires an in-depth analysis to understand the causal relationships and impacts on neonatal health fully.

The higher risk of infection at birth for children who will develop BRUEs during the period of SARS-CoV-2 spread could indicate an increase in exposure to pathogens in the perinatal period, possibly due to variations in hospital practices and increased newborn exposure. The fact that a symptomatic infection during an episode of BRUE was less likely during the period of SARS-CoV-2 spread could reflect a change in the causes of BRUEs or diagnostic practices. This is consistent with significant variations in common respiratory and bacterial diseases globally caused by the SARS-CoV-2 pandemic [22].

Lockdown measures and social distancing have influenced the incidence of diseases [23], slowing down the circulation of pathogens and the development of immunity in the population. This could have contributed to the lower likelihood of fever during a BRUE episode, reflecting a change in the causes of BRUEs or children’s immune responses. Infections from respiratory viruses, such as influenza and respiratory syncytial virus, have decreased significantly at the beginning of the pandemic and have continued to vary during the successive waves of SARS-CoV-2 infections [24]. This could have influenced the average minimum SpO_2_, which was less severe or higher during the SARS-CoV-2 spread, indicating a general improvement in the therapeutic management of pathogens in patients with BRUEs.

A study highlighted that respiratory tract infection symptoms were more frequent in the group with BRUE recurrences than in the group without repetitions (44% vs. 14%, *p* = 0.005). The multivariate analysis confirmed that respiratory tract infection symptoms represent independent risk factors for the recurrence of BRUEs (OR, 5.02; 95% CI, 1.48–16.98) [25]. From the same perspective, the symptoms during BRUE episodes include apnea (73.3%), cyanosis (60.0%), and cough (20.0%). These data further underline the complexity of the interactions that influence the susceptibility and recurrence of BRUEs, requiring a comprehensive evaluation of the multiple factors involved [26]. In the group with recurrence of BRUEs, respiratory tract infection symptoms were more frequent than in the group without repetition (44% vs. 14%, *p* = 0.0055). Respiratory tract infection symptoms were an independent risk factor for the recurrence of BRUEs, with an odds ratio (OR) of 5.02 [25]. The results of our study indicate an O.R. value of 1.922 (C.I. 95% 0.996–3.712; *p* = 0.052) for respiratory symptoms at the diagnosis of BRUEs.

However, a statistically borderline difference (*p* = 0.052) emerged in respiratory symptoms between the pre-COVID period and after the emergence and spread of SARS-CoV-2. The SARS-CoV-2 pandemic increased the risk of respiratory symptoms presenting during a BRUE episode. On the contrary, a statistically significant difference was observed between the two periods for the presence of fever at the time of diagnosis. During the period of SARS-CoV-2 spread, there was a reduction in the likelihood of the risk of presenting rage during a BRUE episode (*p* = 0.046).

These data further support the correlation between infections, particularly of the upper respiratory tract, and the onset of BRUEs [27], confirming the importance of considering respiratory diseases as a significant risk factor for BRUEs in infants. In total, 44.8% of infants had a cause of BRUEs associated with respiratory diseases, confirming a substantial link between respiratory infections and BRUEs [28]. According to one study, most infants with SARS-CoV-2 infection were asymptomatic or had mild symptoms, were generally left to breathe spontaneously, and had a good prognosis [29]. These results underline the need for a thorough clinical evaluation, as respiratory diseases have emerged as a significant risk factor for BRUE in infants, paving the way for careful consideration of the implications of potential hypoxia related to these conditions.

Infected infants, even if asymptomatic or with mild symptoms, may maintain variable levels of hypoxia before manifesting evident symptoms. Infants may act as silent carriers of the virus, and chronic airway inflammation could contribute to the remodeling and thickening of the same [30]. Our study shows a statistically significant difference in the minimum SpO_2_ (%) in children with BRUEs between the pre-COVID-19 period and the one following the emergence and spread of SARS-CoV-2. In particular, the increase in the average minimum SpO_2_ (%) seems to correlate with a higher likelihood of belonging to the period after the spread of SARS-CoV-2.

This data integrates with the variation in the incidence of apneas in infants with RSV infection, which can vary significantly, ranging between 10% and 26% [31]. Although viral infection is not commonly associated with BRUEs, thorough microbiological investigations could clarify the aetiology of such events, even without manifest signs of respiratory disease, thus contributing to the overall understanding of the underlying factors [8,32]. In addition, factors such as symptoms of respiratory infection increase the likelihood of BRUEs [27].

Our study has some limitations. The potential for selection bias is an inevitable consideration given the retrospective nature of our study and the reliance on medical records from newborns admitted to our hospital for BRUEs. Another concern regarding selection bias relates to the sample size and the representativeness of our study population compared to the general population of newborns affected by BRUEs [33]. Since our sample consists of newborns admitted to our hospital for BRUEs, it may not accurately represent all newborns with BRUEs in the general population. This is particularly true if specific demographic or clinical characteristics influence hospital admission.

Additionally, our sample may not fully capture the geographical or socio-economic variations in the prevalence and management of BRUEs, potentially limiting the generalizability of our study’s results [33]. Furthermore, our study did not account for other factors that could influence the outcomes, such as the severity of SARS-CoV-2 infection or the patients’ underlying health conditions. Further research is necessary to validate our findings and to understand the mechanisms underlying these results. Studies with larger and more representative samples of the general population are needed. Moreover, research should explore the association between the variables studied and other clinical outcomes, such as disease duration, the need for hospitalization, or mortality.

In summary, based on our analysis, we have found that although there were cases of SARS-CoV-2 infection during pregnancy, they represented a small proportion of the total observed cases. Therefore, some enrolled children may have escaped diagnosis of COVID-19 infection because they were not tested as they were asymptomatic outside the time when they developed BRUEs. Thus, the results would suggest that COVID-19 may not have a significant epidemiological impact on the development of BRUEs.

Other viruses [34], such as RSV and rhinovirus associated with BRUEs, may be found as pathogens in patients with BRUEs during or before the pandemic, but the number of cases has been reduced. Specifically, only a small percentage of cases involved COVID-19 detection in BRUE cases [9,35], indicating a relatively low frequency compared to other factors contributing to BRUEs.

Our findings highlight intriguing patterns regarding the risk of infection and symptomatology during the spread of SARS-CoV-2. Additionally, variations in the likelihood of asymptomatic infection, respiratory symptoms, fever, and SpO_2_ levels have been observed during BRUE episodes in the context of the global spread of SARS-CoV-2.

Changes in the environment and medical practices may have contributed to the observed differences in infection rates and respiratory symptoms before and after the onset of the COVID-19 pandemic [36]. During the SARS-CoV-2 spread period, measures such as social distancing, widespread mask usage, and other preventive practices may have contributed to reduced exposure to infections, including the risk of contracting viruses during pregnancy and childbirth [37]. On the other hand, there may have been increased medical attention on neonatal infections and respiratory symptoms, leading to more significant detection and recording of such events. Additionally, the interaction between the maternal immune system and virus exposure may have had complex effects on the risk of infection during pregnancy [38,39].

## 5. Conclusions

The results of this study have highlighted that, during the spread of SARS-CoV-2, mothers of children with BRUEs showed a lower risk of infection during pregnancy, while the infectious risk at birth of children who will develop BRUEs was higher. During the same period, asymptomatic infection during BRUEs was less likely, while respiratory symptoms and fever were more frequent and less likely, respectively. Additionally, the minimum oxygen saturation was less severe or higher during the spread of SARS-CoV-2. These findings could have significant clinical implications for managing children with BRUEs during the COVID-19 pandemic. For example, they may suggest the importance of closely monitoring respiratory symptoms and fever in children with BRUEs during the virus spread. However, further research may be necessary to confirm and fully understand these associations.

## Figures and Tables

**Table 1:** Overview of dichotomous variables related to infections and respiratory conditions during pregnancy and BRUEs. The table displays the number of cases and the percentage for each variable, including specific diseases, respiratory symptoms, detection of viruses, and the outcome of home monitoring.

Dichotomous Variable	Total No. of Cases	% (of 454 Cases)
Infections in pregnancy	30	6.6
CMV Infection in Pregnancy	0	0.06
SARS-CoV-2 infection in pregnancy	17	3.7
Infectious risk at birth	61	13.4
Ongoing symptomatic infection in BRUE	344	75.8
Ongoing respiratory symptoms in BRUE	53	11.7
COVID detected in BRUE	2	0.4
RSV Detected in BRUE	3	0.7
Rhinovirus detected in BRUE	4	0.9
GBS detected in BRUE	2	0.4
Ongoing fever in BRUE	135	29.7
Pathologic breathing during BRUE	336	74
Periodic breathing during BRUE	146	32.2
Desaturations detected with Gatemed (yes)	249	54.8
Short apnea (yes)	347	76.4
Long apnea (yes)	2	0.4
O_2_ therapy (yes)	52	11.5

Legend: BRUE, Brief Resolved Unexplained Events; CMV, cytomegalovirus; GBS, group B streptococcus; Gatemed, portable recording device; RSV, Respiratory syncytial virus.

**Table 2:** Comparative overview of continuous variables between the pre-COVID-19 period and the onset of the SARS-CoV-2 pandemic. The table displays each variable’s mean and standard deviation (S.D.), including the total number of patients, birth weight, age at the BRUE, and the duration of home monitoring. Additionally, it provides the asymptotic significance value for a two-way test.

Binary Logistic Regression	B	S.E.	Wald	*p*	Exp(B)	95% C.I. per EXP(B)
Dependent variable (pre-COVID-19 = 0, SARS-CoV-2 pandemic = 1) *						
Infection in pregnancy	−1.839	0.551	11.157	0.001	0.159	0.054–0.468
Infectious risk at birth #	0.783	0.302	6.747	0.009	2.189	1.212–3.954
Ongoing symptomatic infection in BRUE	−0.413	0.245	2.847	0.092	0.662	0.409–1.069
Ongoing respiratory symptoms in BRUE	0.654	0.336	3.789	0.052	1.922	0.996–3.712
Ongoing fever in BRUE	−0.465	0.232	4.000	0.046	0.628	0.398–0.991
SpO_2_ min (%)	0.055	0.015	13.467	<0.001	1.057	1.026–1.089
Dependent variable (Symptomatic infection during BRUE, 1 = yes) **						
Pre-COVID-19 = 0, spread of SARS-CoV-2 = 1	−0.464	0.234	3.926	0.048	0.629	0.397–0.995
Infection in pregnancy	1.646	0.406	16.431	0.000	5.184	2.339–11.487
Age (days)	−0.004	0.001	8.414	0.004	0.996	0.994–0.999
AHI (events/hour)	−0.019	0.008	5.339	0.021	0.981	0.965–0.997
Dependent variable (Respiratory symptoms during BRUE, 1 = yes **						
Pre-COVID-19 = 0, spread of SARS-CoV-2 = 1	0.601	0.332	3.273	0.070	1.824	0.951–3.498
Infection in pregnancy	−1.213	0.500	5.900	0.015	0.297	0.112–0.791
Age (days)	0.009	0.002	30.256	0.000	1.009	1.006–1.012
SpO_2_ min (%)	−0.033	0.017	3.774	0.052	0.967	0.935–1.000
Dependent variable (Fever during BRUE, 1 = yes) **						
Pre-COVID-19 = 0, spread of SARS-CoV-2 = 1	−0.504	0.238	4.487	0.034	0.604	0.379–0.963
Age (days)	0.010	0.002	47.178	0.000	1.010	1.007–1.013
SpO_2_ min (%)	−0.030	0.015	4.146	0.042	0.971	0.943–0.999

# Positive vaginal swab for GBS, PROM > 18 h, maternal fever in labor, foul-smelling stained amniotic fluid. * Variables entered in phase 1: birth weight (grams); infections during pregnancy (yes = 1, dichotomous variable); infectious risk at birth (yes = 1, dichotomous variable); symptomatic infection (yes = 1, dichotomous variable); respiratory symptoms at the event (yes = 1, dichotomous variable); fever at the event (yes = 1, dichotomous variable); pathological breathing (yes = 1, dichotomous variable); AHI (events/hour, continuous variable); minimum SpO_2_ (%, continuous variable); average SpO_2_ (%, continuous variable). ** Variables entered in phase 1: birth weight (grams); age (days, continuous variable); infection during pregnancy (1 = yes, dichotomous variable); infectious risk at birth AHI (events/hour, continuous variable); minimum SpO_2_ (%, continuous variable); average SpO_2_ (%, continuous variable).

## Data Availability

The data presented in this study are available in this article (and Supplementary Material).

## Supplementary Materials

The following supporting information can be downloaded at: https://www.mdpi.com/article/10.3390/life14030392/s1, Table S1: Comparative overview of continuous variables between the pre-COVID-19 period and the onset of the SARS-CoV-2 pandemic.
