# Supplementary material for: Effects of the COVID-19 Pandemic on Brief Resolved Unexplained Events (BRUEs) in Children: A Comparative Analysis of Pre-Pandemic and Pandemic Periods
Source: Life (Basel). 2024 Mar 15;14(3):392. doi: 10.3390/life14030392 (PMC10971196; doi:10.3390/life14030392)
Supplement: Supplementary file 1 [file life-14-00392-s001.zip › life-2851388-supplementary.pdf]

life

MDPI

Supplementary Materials

Table S1. Comparative overview of continuous variables between the pre-COVID-19 period and the onset of the SARS-CoV-2 pandemic. The table displays each variable's mean and standard deviation (S.D.), including the total number of patients, birth weight, age at the BRUE event, and the duration of home monitoring. Additionally, it provides the asymptotic significance value for a two-way test.

Continuous Variable	Mean ± S.D.	Mean S.E.	Mean ± S.D.	Mean S.E.	Sign. Asymptotic
	pre-COVID-19		Start-SARS-COV-2		
Total number of patients (% males)	186 (51.6)		268 (42.5)	-	0.056
Birth weight (grams)	3,094 ±609	44.6	3,072 ±643	39.3	0.588
Age (days) at the event BRUE	101.3 ±100.6	7.4	98.5 ±84.3	5.2	0.764
Duration of home monitoring (days)	123.6 ± 148.0	10.9	105.9 ± 99.6	6.1	0.128

Legend: BRUE, Brief Resolved Unexplained Event; S.D., standard deviation; S.E., standard error.
